# Genome-Wide Identification and Characterization of Olfactory Receptor Genes in Chinese Perch, *Siniperca chuatsi*

**DOI:** 10.3390/genes10020178

**Published:** 2019-02-25

**Authors:** Li-Yuan Lv, Xu-Fang Liang, Shan He

**Affiliations:** 1 College of Fisheries, Chinese Perch Research Center, Huazhong Agricultural University, Wuhan 430070, China; llyuan.2009@163.com (L.-Y.L.); heshan@mail.hzau.edu.cn (S.H.); 2 Freshwater Aquaculture Collaborative Innovation Center of Hubei Province, Key Lab of Freshwater Animal Breeding, Ministry of Agriculture, Wuhan 430070, China

**Keywords:** Chinese perch (*Siniperca chuatsi*), olfactory receptor, expression profiles, olfaction, adaptive evolution

## Abstract

Olfaction, which is mediated by olfactory receptor (OR) genes, is essential in the daily life of fish, especially in foraging. However, Chinese perch (*Siniperca chuatsi*) is believed to prey with reliance on vision and lateral sensation, but not on olfaction. Therefore, understanding the evolutionary dynamics of the Chinese perch OR repertoire could provide insights into genetic evidence for adapting to a decreasing reliance on olfaction. Here, we reported a whole-genome analysis of the Chinese perch OR repertoire. Our analysis identified a total of 152 OR genes, including 123 functional genes and 29 pseudogenes, and showed their genomic organization. A phylogenetic tree was constructed, and the phylogenetic relationships of teleosts ORs was illustrated. The dN/dS (global ratios of non-synonymous to synonymous) analysis demonstrated that OR groups all appeared to be under purifying selection. Among the five Percomorpha fishes, Chinese perch only had 22 subfamilies, suggesting a decrease in OR diversities. The species-specific loss of subfamily 56 and 66 in Chinese perch, of which the genes belonged to subfamily 66, were orthologs of OR51E2, which recognized the plant odorant β-ionone, indicating that extremely piscivorous fish which might lose those receptors responded to plant-related odors. Finally, the expression profiles of OR genes in the olfactory epithelium at different developmental stages were investigated using RNA-seq data. From the aforementioned results, the evolution of the OR repertoire may be shaped by the adaption of vision-dependent specializations for foraging in Chinese perch. The first systematic study of OR genes in Chinese perch could provide valuable genomic resources for the further investigation of olfactory function in teleosts.

## 1. Introduction

It is well-known that animals have five basic senses (olfaction, vision, gustation, hearing, and touch), which are thus of fundamental importance in the lives of animals. However, one or more of the five senses could be absent in some species, such as whales, which lack the specific olfactory functions that induce innate avoidance behaviors against odors of predators and spoiled foods [1]. The sense of smell, or olfaction, is mediated by olfactory receptors (ORs), which are mainly responsible for the perception and detection of tens of thousands of chemical odors. Olfaction is essential for fish survival, due to its action in recognizing appropriate food, relatives, offspring, habitats, and predator avoidance [2,3,4,5]. Besides, the exquisite expression of OR genes in non-olfactory tissues, such as in the liver, heart, ovaries and testis, also indicates that OR genes probably display some other functions [6,7,8,9]. 

ORs are G-protein-coupled receptors (GPCR) with seven-transmembrane domains, encoded by OR genes [10]. The OR genes make up one of the largest gene superfamilies in most animal genomes. With more and more genomes being successfully sequenced, investigations have been conducted to explore OR subgenomes in a variety of species, including lamprey, zebrafish, fugu [11], frogs [12], birds [13] and mammals [14,15]. The results revealed that the numbers of OR repertoires varied dramatically, ranging from ~30 in penguins, to 1948 in the African elephant [13,16]. Previous studies on human, rodents, and birds demonstrated that not only the number of total OR genes, but also the proportions of pseudogenes were considered to reflect upon their ability to smell [17]. Rodents have to rely more on smell to forage and communicate than humans (percentage of pseudogenes = pseudogenes/total number of OR genes: rat = 508/1767, human = 414/802), who have obtained trichromatic color vision, as well as birds [13,17,18,19]. Birds have a lower number of intact ORs, suggesting a reduced degree of reliance on olfaction compared to most mammals, which is in agreement with the common opinion that most birds are primarily visual-dependent animals [20]. Hence, comprehending OR subgenomes would be significant for understanding the tradeoff between olfaction and vision, shaped by adaptations of foraging behavior.

The classification of OR genes is intricate. Vertebrate OR genes have recently been divided into two major types, which include 10 groups in total, type I and type II. Among them, six groups, α, β, γ, δ, ε, and ζ, belong to type I, and four groups, η, θ, κ, and λ, belong to type II. It is worth mentioning that groups θ, κ, and λ are expected to be likely to be non-OR genes, because they are not found to be expressed in olfactory tissues [2,21]. Fish OR genes have been prevailingly classified into β, δ, ε, ζ, and η, and they have almost completely lost α and γ, which are assumed to recognize air-borne molecules.

Chinese perch (*Siniperca chuatsi*), an outstanding commercial aquaculture species in China, has a delicious taste with high nutritional value. In addition, Chinese perch is an extreme piscivore. Since the fry start feeding, they only accept live prey. It has been long believed that they have had a functional degeneration of smell for foraging, based on physiological and behavioral studies, as they primarily rely on vision and touch [22] As yet, at the molecular level, there is no survey on the genetic factors mediating olfactory responses in Chinese perch. In this study, we first analyze a nearly complete OR repertoire in the Chinese perch genome. The chromosome localization, genome composition, phylogeny, conserved motifs, and expression profiles of OR genes were systematically analyzed, providing a basis for further investigation of the functions of OR genes. The results will contribute to understanding the adaptable evolution of olfaction in piscivorous fish. 

## 2. Material and Methods

### 2.1. Genome-Wide Identification of OR Genes in the Genomes of Chinese Perch and Other Vertebrates

The OR repertoires from three vertebrates, including zebrafish (*Danio rerio*), stickleback (*Gasterosteus aculeatus*), and fugu (*Takifugu rubripes*) were obtained from the literature [11]. The above known full-length OR amino acid sequences were used as query sequences to conduct TBLASTN against the following genomes from four fishes. The draft genomes of spotted gar (*Lepisosteus oculatus*) and tongue sole (*Cynoglossus semilaevis*), were retrieved from NCBI [23]. European seabass (*Dicentrarchus labrax*) genome was retrieved from the seabass genome website [24]. The Chinese perch (*Siniperca chuatsi*) draft genome was sequenced using Single Molecule Real-Time (SMRT). The genomic contigs containing the OR gene cluster are provided in Supplementary data 1. To identify OR repertoires as completely as possible, we followed a standard protocol as described previously, with slight modifications [12]. 

Firstly, a TBLASTN search was administered to identify the candidate OR genes with an e value cut-off of 10. The best hits with the criteria of the lowest e-value and longest alignment were retained for the next step analysis. For candidates without start/stop codons, we further searched 1000 bp upstream and 1000 bp downstream, to find putative start codons or stop codons. Secondly, all of the candidate OR genes were compared back to the NCBI non-redundant database (BLASTX). The candidate OR genes that matched the best with corresponding non-OR GPCR genes were discarded. Then, the remaining candidate OR genes were systematically classified into three categories: “functional genes” if they were at least 250 amino acids in size with no interrupting stop codons or frameshifts within the ORFs, and that could code for seven TM domains; “pseudogenes” if they were at least 250 amino acids in size, but there were stop codons and/or frameshifts within the ORFs; and “truncated genes” without start/stop codons or both, but well matched to the known ORs. Lastly, whole new functional OR genes were translated into amino acid sequences, using EMBOSS explorer [25]. 

### 2.2. Classification of OR Genes

To classify all of the newly-retrieved OR genes into their respective OR groups, we constructed an unrooted neighbor-joining (NJ) tree, using known OR genes belonging to groups α–λ from zebrafish, stickleback, and fugu with 1000 bootstraps by MEGA7.0. The NJ tree helped us to assign each OR gene to the closest known OR gene, according to the best similarity, with high reliability. The accuracy of the assignment was monitored, by assigning the known zebrafish OR genes into a conducted NJ tree with each of the known OR genes being assigned exactly to each group.

### 2.3. Phylogenetic Analysis of OR Genes

The translated amino acid sequences of the OR genes in Chinese perch, spotted gar, zebrafish, tongue sole, stickleback, fugu, and European seabass were aligned using MAFFT 7, with auto strategy parameters. Phylogenetic analyses were performed using IQ-TREE [26] with Maximum Likelihood (ML) approaches, based on a JTT+F+G10 model of amino acid evolution. The unrooted phylogenetic tree was generated with 1000 rounds of bootstrapping, and better visualized using iTOL [27]. 

### 2.4. dN/dS Analysis

The global ratios of non-synonymous to synonymous mutations (ω = dN/dS) were calculated with nucleotide sequences, using the Datamonkey web server [28]. To measure the natural selection pressure, we used the fixed effects likelihood (FEL) described in reference [29]. FEL uses the entire alignment to infer the model parameters shared by all codon sites (e.g., branch lengths) and then fits the dN and dS rates individually. All of the genes (both orthologs and paralogs) within a single OR group were aligned for FEL analysis. Additionally, the average dN/dS ration was obtained for the entire group.

### 2.5. Analysis of Protein-Conserved Motifs

To identify the conserved motifs in the predicted OR amino acid sequences, sequence logos were generated from the alignment of functional OR protein sequences, with the program Multiple Expectation Maximization for Motif Elicitation v.4.12.0 (MEME) [30]. Only the top five conserved motifs were identified, with the motif length ranging from five to fifty. NetNGlycserver was used for predicting the potential *N*-glycosylation sites [31]. It was considered to be a positive *N*-glycosylation site, with a “potential” value of above 0.5, and an aboard agreement of “++” or higher.

### 2.6. Detection of Chinese Perch-Specific Gained and/or Lost OR Genes

Multiple sequence alignment was performed with multispecies OR protein sequences from five Percomorpha fishes (Chinese perch, tongue sole, stickleback, fugu, and European seabass) using Clustal Omega, in order to group them based on the sequence identity. The cutoff value for a subfamily was 60% identity at the level of the protein sequence. 

### 2.7. Expression Profile Analysis of OR Genes

To further characterize the different temporal gene expression patterns of the OR gene family, we analyzed RNA sequencing (RNA-seq) data. Transcriptome sequencing datasets were deposited in the BioProject ID PRJNA507831, which was used to perform RNA-seq of the olfactory epithelium (OE) tissues obtained from two developmental stages, including 30 d post-hatch (30 dph) and 1-year-old (adult) *S. chuatsi*. The OE tissues were collected and immediately frozen in liquid nitrogen for RNA extraction. All samples were replicated three times. We quantified the gene expression levels based on their fragments per kilobase of exon per million read-mapped (FPKM) values, using Cufflinks with default parameters [32].

## 3. Results

### 3.1. Genomic Organization of the Chinese Perch OR Repertoire

**Figure 1:**
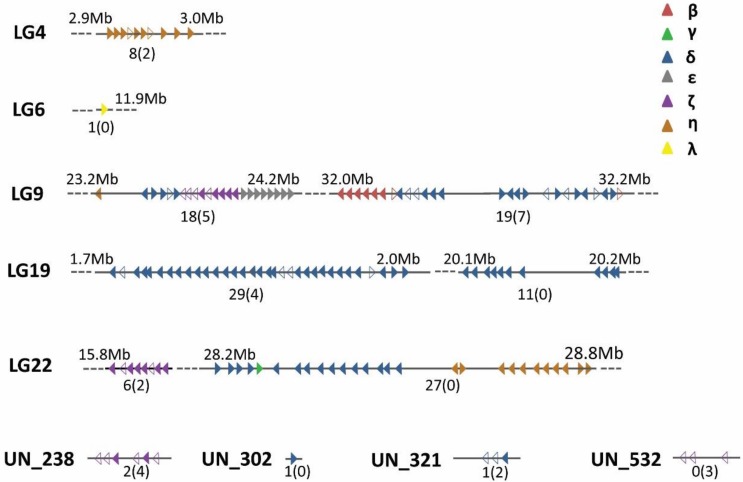
Chromosomal distribution of Chinese perch OR genes. The majority of Chinese perch OR genes were organized in eight clusters on five chromosomes. The other 13 OR genes were located on four scaffolds. The position of each cluster is shown above the chromosomes, in Mb. The numbers of functional genes and pseudogenes in each cluster are shown below the chromosomes/scaffolds. OR genes are depicted as filled triangles (functional genes) and hollow triangles (pseudogenes). Triangles pointing to the right mean the + strand; triangles pointing to the left mean the − strand; the OR gene distance is drawn to scale. Genes are colored according to their group.

Similar to the previous study on the identification of OR genes from the teleost genome, the known OR genes from NCBI were used for searching the whole repertoire of OR genes in the Chinese perch genome (unpublished data). The Chinese perch genome contains 152 OR genes, including 123 functional genes and 29 pseudogenes (Table 1). The five chromosomes contained 1 to 49 OR genes, and LG19 had the largest number of functional genes (*n* = 40), followed by LG9 (*n* = 37) and LG22 (*n* = 33). In addition, the two highest proportions of pseudogenes were 24% and 20% on LG9 and LG4, respectively. Based on the physical positions of the 152 OR genes, 139 were densely mapped onto five chromosomes, whereas the remaining 13 genes were located on the unmapped scaffolds, and the relative distributions were illustrated in Figure 1. Moreover, the OR genes were considered to group into clusters if the gene sequences were more than one megabase (Mb) apart. The number of OR genes at a single cluster ranged from 1 to 33 (Figure 1 and Supplementary data 2). More detailed information about the distributions and sequence information of the OR repertoire in the Chinese perch genome are summarized in Supplementary data 2.

### 3.2. Classification of the OR Gene Repertoire

OR genes were the largest gene superfamily in the vertebrates genome. Niimura and Nei [12] classified the vertebrates OR genes into Type I, with six groups (α–ζ), and Type II, with five groups (η–λ). The genes of groups θ, κ, and λ were phylogenetically nested within the vertebrate OR genes, but they apparently did not belong to OR genes [11]. To better understand the different functions among the different groups, the OR genes required systematic classification, based on their structural similarities. The queried Chinese perch OR genes were classified into different groups, according to the results of the NJ tree (not shown) conducted with the known OR genes, as described in the methods. The numbers of OR genes belonging to each group are shown in Table 2 (details also in Supplementary datas 2 and 3). OR genes belonging to groups α and γ were mostly absent in fishes. However, there were still 37 functional genes belonging to groups α and γ in spotted gar, an ancient fish. Group δ was the largest group, in which the numbers of members ranged from 55 to 112. Seabass and Chinese perch both had the largest numbers of functional genes in group δ, n = 79 and 74, respectively. With the exception of spotted gar, the second-largest group was η. It was interesting that there were 13 pseudogenes belonging to group ζ in Chinese perch, though the other fish only had less than four pseudogenes. 

### 3.3. Phylogenetic Analysis of the OR Gene Repertoire

**Figure 2:**
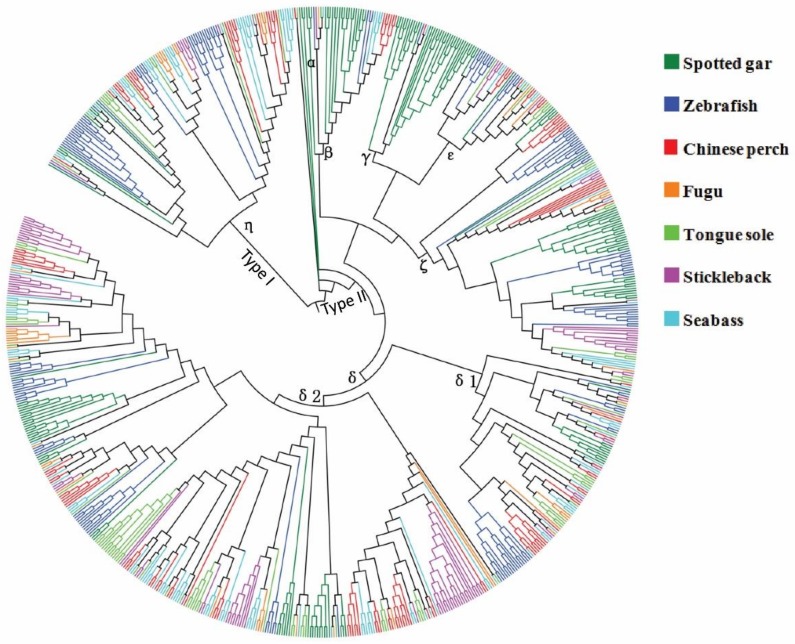
A phylogenetic tree of OR functional genes from Chinese perch and six other teleost genomes (n = 808) (see also Supplementary datas 2 and 3). Legends are indicated on the upper left-side of the figure. Groups named α to η are signed on the branches. All of the major clades have bootstrap values greater than 90%.

To examine the evolutionary relationships among the teleost OR genes, we used MAFFT and the IQ-TREE program to align and to construct a phylogenetic tree with 808 OR functional genes in total. A phylogenetic tree of OR genes was performed as shown in Figure 2. The results showed a good agreement with the evolutionary relationships among the selected species. Following the phylogenetic analysis, Chinese perch OR genes had a closer relationship with those of seabass, stickleback, fugu, and tongue soles, which all belonged to Percomorpha, compared to zebrafish and spotted gar. Moreover, the OR genes were clearly classified into two major clades, type I and type II. Of note, non-OR genes were nested into a clade of type II. According to the nomenclature of the OR genes, six groups were identified in the Chinese perch OR repertoire, including groups β, γ, δ, ε, ζ, and η. Five subclades with high bootstrap values (> 85%) were assigned to group δ (δ1 and δ2), and two subclades to group η. 

### 3.4. Evolution of dN/dS Ratio

**Figure 3:**
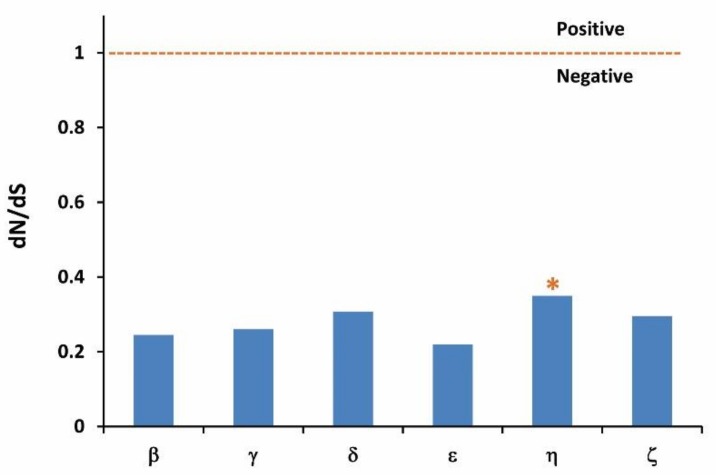
Selection pressure imposed on the OR groups. The asterisk (*)-indicates that group η exhibits the highest dN/dS ratio.

The results of the dN/dS analysis are shown in Figure 3. The global ratios of dN/dS below 1.0 suggest a negative selection pressure, and higher than 1.0 indicate positive selection [33]. The global ratios of dN/dS are all below 1.0 for OR groups in Chinese perch, which extend from 0.2 for group ε, to 0.35 for group η.

### 3.5. Patterns of Conserved Motifs for OR Genes

**Figure 4:**
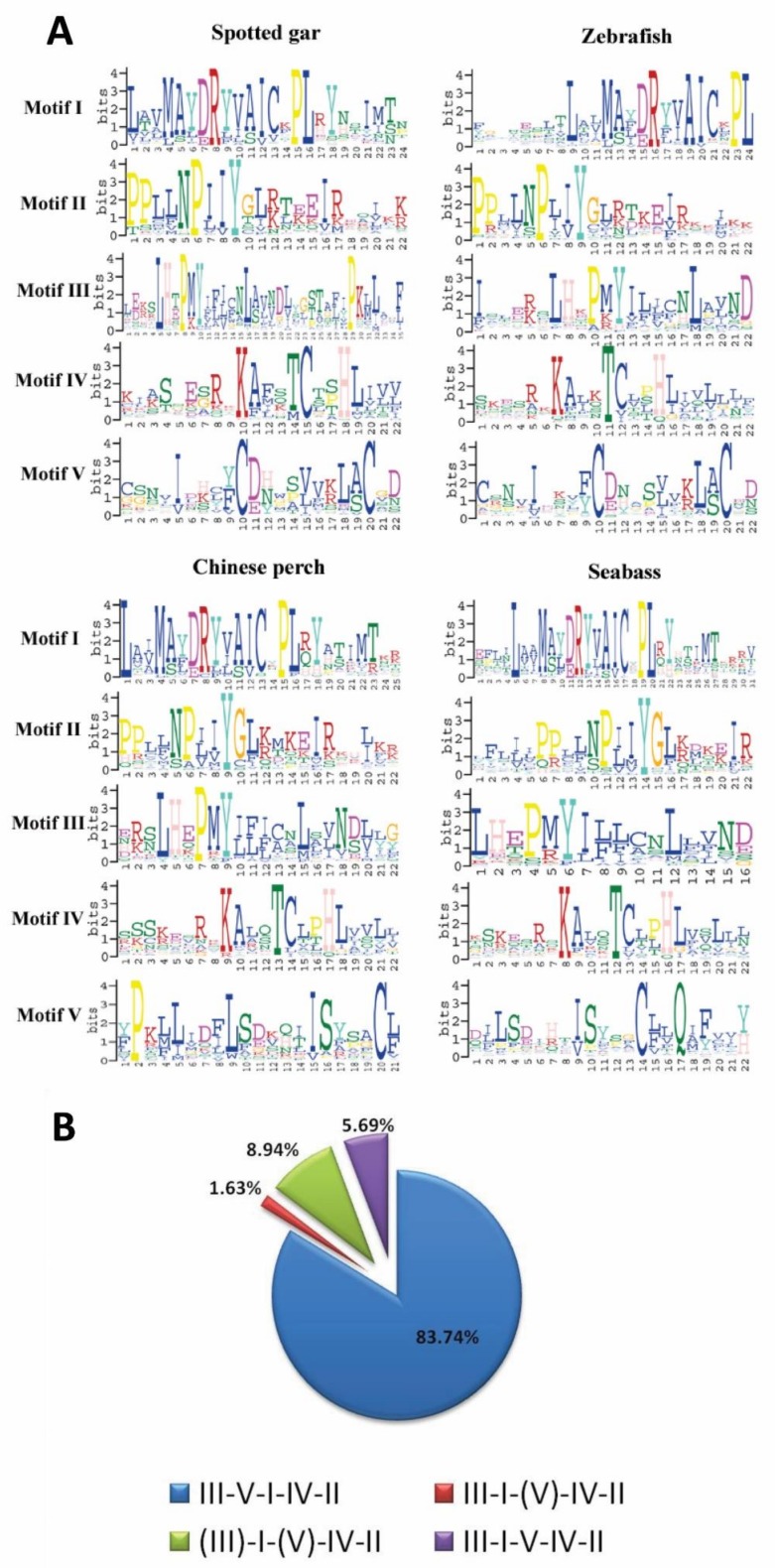
Logo representation of the five best conserved motifs identified for teleost OR genes, and the frequency of sequences with or without these motifs in Chinese perch. (**A**) Sequence logos of the conserved motifs, as the degree of conservation, was represented by the height of the amino acid code. (**B**) Proportional distribution of the total functional OR protein sequences, identified by their OR motif-containing patterns in Chinese perch. The motifs within parentheses were absent.

To characterize the conserved motifs of the OR amino acid sequence, the five most conserved motifs for four species (spotted gar, zebrafish, Chinese perch and seabass) were identified by the MEME program. As shown in Figure 4A, the first four motifs were strikingly similar among teleosts. However, the fifth motif did not show extremely clear patterns for these fishes, which were common to cichlids [34]. For a better understanding of the structural diversity of Chinese perch OR genes, we calculated the number of conserved motifs for each OR functional gene. The gene structure analysis suggested that 83.74% (103/123 genes) of OR functional genes all contained the five motifs, indicating a high conservation of OR sequences. The remainder (20/123 genes) were missing motif III and/or motif V, which all belonged to group η (Figure 4B). In addition, a conserved *N*-linked glycosylation site was detected in all OR functional genes, but no signal peptide was present (Supplementary data 7). The presence of the same conserved motifs and the N-linked glycosylation sites implied that the functions of the OR genes were similar at the protein level.

### 3.6. Potential Species-Specific OR Genes in Chinese Perch

To explore the evolutionary features of OR genes among the five Percomorpha fishes (Chinese perch, European seabass, tongue sole, fugu, and stickleback), we combined 499 OR functional genes, and performed clustering according to their amino acid sequence identity (Supplementary data 4). Using the criteria for a cutoff of higher than 60% sequence identity, the numbers of OR families and subfamilies ranged from 17 to 25 and 22 to 45 in each fish, separately. However, only 80 subfamilies were found among the five Percomorpha fishes (Supplementary datas 5 and 6). A proportion of 21% (17/80 subfamilies) of OR subfamilies were common to four species. Moreover, we observed that Chinese perch had only seven species-specific genes belonging to subfamily 60, which was much less than tongue sole (50 genes) (Table 3). The numbers of subfamilies, which were specific to Chinese perch, European seabass, tongue sole, fugu, and stickleback, were 1 (seven genes), 9 (13 genes), 18 (50 genes), 7 (9 genes), and 2 (22 genes), respectively (Table 3 and Supplementary data 5). 

### 3.7. Expression Profile Analysis of OR Genes at Different Developmental Stages

**Figure 5:**
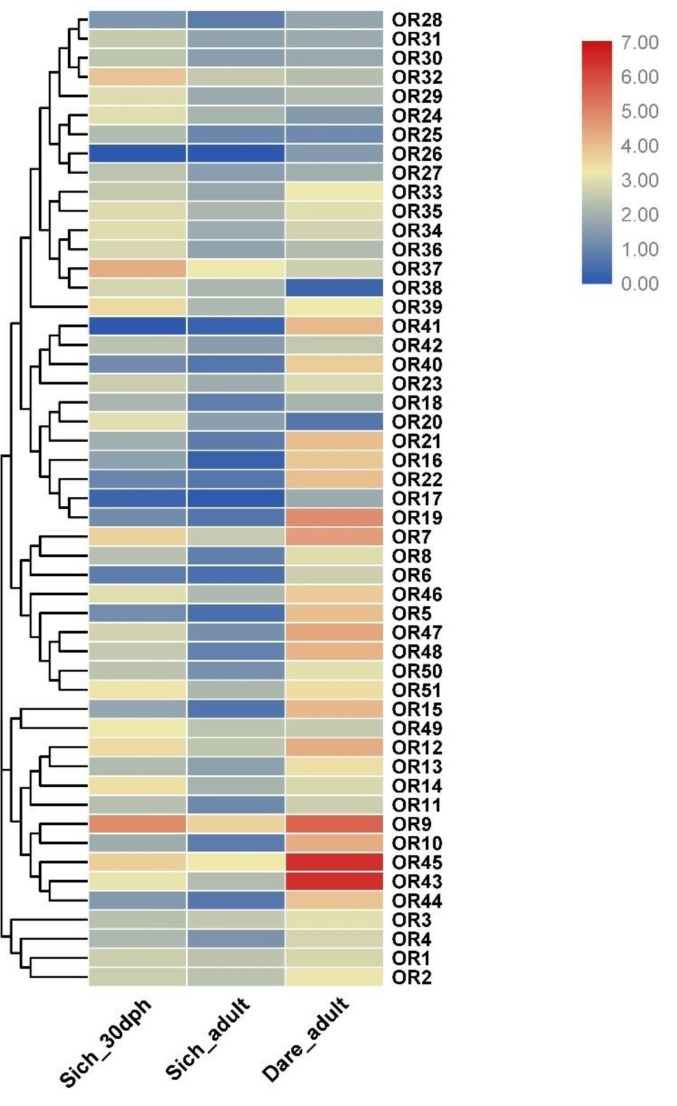
Expression profiles of OR genes in the olfactory epithelium. Heat map showing the expression levels of OR genes in two stages of 30 dph and adult Chinese perch, and the adult zebrafish. As shown in the bar to the right of Figure 5, gene transcript abundance is represented by different colors in the map. The FPKM (fragments per kilobase of exon model per million mapped fragments) values of the OR genes are presented in Supplementary data 8.

To further understand the expression patterns of the OR genes, we analyzed the RNA-seq data of *S. chuatsi* OE. The results showed that out of 152 OR genes, 64 OR genes displayed differential expression at in 30 dph, and in adult OE tissues (Figure 5 and Supplementary data 8). In order to confirm the hypothesis that Chinese perch relies more on vision and touch senses than on smell, we compared the expressions of orthologous OR genes between adult Chinese perch and zebrafish (PRJEB4464 [35]). Figure 5 shows the expression profile of 51 orthologous OR genes, which were expressed both in adult Chinese perch and in zebrafish. Notably, the expression levels of most OR genes in 30 dph OE were higher than in adults, except for OR3 (SichOR6.1-kappa), OR26, (SichOR19.10-delta) and OR41 (SichOR19.43-delta). We also found that compared with the average expression values of 12.580 FPKM in adult zebrafish, OR genes in Chinese perch displayed much lower levels of expression, at 30 dph with 5.765 FPKM, and adults with 2.510 FPKM respectively. The expression levels of the same gene differed between different growth stages, suggesting that the genes play different roles at specific times.

## 4. Discussion

The olfactory system is essential for fish to avoid aggression by predators or enemies, to search for suitable food and to select appropriate sexual partners. Therefore, olfaction is a vital sense throughout the fish’s entire life. However, Chinese perch prey on live prey, fish relying chiefly on vision, but not olfaction. Taken together, it is a worthy question of how the evolutionary dynamics of OR repertoire evolved in the Chinese perch genome. With the aid of genome sequencing, researchers have found striking variations in the numbers of OR genes among vertebrates, indicating that the olfaction machinery in animals was strongly influenced by natural selection [36]. Here, we firstly reported the identification of the OR gene repertoire in the genome of the Chinese perch.

### 4.1. Characterization of OR Genes in Chinese Perch

To obtain a OR repertoire that is as complete as possible, we refer to a strategy that was used by the literature [12] and [34], for an analysis of Chinese perch, European seabass, and tongue sole. We accomplished a thorough TBLASTN search for OR sequences corresponding to a set of certain OR genes retrieved from the reference [11]. As with the criteria of cichlid OR gene searching, we also did not discard slightly positive hits of less than 700 nucleotides. Then, we obtained all hits, and checked every candidate gene or gene fragment, whether intact or not. As a result, we identified a total of 152 OR genes in the Chinese perch genome, of which 122 were functional genes, which were similar to large yellow croaker [21]. As we have seen, the number of OR genes varied greatly in each species. Spotted gar and zebrafish possessed the largest repertoire sizes of the functional OR genes, respectively. Also, the fugu had the smallest repertoire size of the OR genes, which was only half that of zebrafish. The repertoires of tetrapods OR genes were much larger than those of teleosts. For instance, more than 1000 functional OR genes were retrieved in elephant and pig, ~800 in frog and ~380 in human [11,16,37]. To some degree, the higher number of OR functional genes indicates a heavier reliance on olfaction [2,13]. As such, our genetic evidence suggested that Chinese perch have not completely lost their sense of smell. We speculated that the capacity for olfaction in Chinese perch might be similar to that of Europe seabass, which has an analogous mode of foraging behavior [38]. 

Previous studies have demonstrated that OR genes are arranged tightly into clusters in vertebrate genomes [16,39,40,41]. In mammalian genomes, OR genes are distributed widely, residing on 26 chromosomes in cattle [39], and on 18 chromosomes in the mouse [41]. When we characterized the structures of the OR gene clusters in Chinese perch, we did not observe any distinctions or patterns, compared with the zebrafish OR gene clusters [40]. Like zebrafish, we found seven major clusters on four chromosomes in the Chinese perch genome. We were able to describe the genomic locations accurately for more than 90% of OR genes (13 remain unmapped to chromosomes). The OR genes in the same cluster, which were mostly contiguous and tightly adjacent, usually share the same transcriptional orientations, suggesting tandem duplication as a mechanism of expansion within a cluster, which involves a group of paralogous genes that plays a similar recognition function, and that are very likely to be regulated through the same pattern. This was consistent with previous views that tandem duplication is a principal type of duplication in teleosts [42], and they might contribute to their adaptive evolution [43,44]. 

### 4.2. Phylogeny of OR Genes in Chinese Perch

Before amphioxus appeared, OR genes originated prior to the advent of chordates [11]. The divergence of type I and type II OR genes antecedently generated the divergence of jawless and jawed vertebrates. Ancient OR genes diverged into seven groups (α, β, γ, δ, ε, ζ, and η) during the time close to the bifurcation of jawless and jawed fishes. With the development of teleost and tetrapod evolution, teleosts evolved most of their fish-like OR groups (β, δ, ε, ζ, and η), which were lacking in tetrapods. The majority of fish-like OR genes were considered for the detection of water-soluble odorants [12]. However, fishes have lost the mammalian-like groups (α and γ) except in the case of coelacanths, which are known as living fossils that exist from the time that all tetrapods emerged [45,46]. It has been suggested that evolutionary traces were the reason for the remaining residua genes of groups α and γ in “ancient” fish. In our research, the same results were observed in spotted gar, whose lineage evolved significantly slower than teleosts [47]. Phylogenetic analysis was performed in order to elucidate the evolutionary relationships of OR genes among seven modern teleosts. We reached a result similar to that of previous conclusions [12,21,40,48]. The phylogenetic relationships of OR genes among the extant fishes were coincident with their evolutionary history [21]. OR genes were more closely related to the five Percomorpha fishes than to spotted gar and zebrafish, in the sense that they are close evolutionary relatives. During the period of speciation, a high percentage of OR genes have been pseudogenized, as shown in Table 2. Up to 50% of group ζ OR genes were pseudogenized, and they may have lost their functionality in Chinese perch, but they do not lack fish-like groups compared to other teleosts, even though the food choices in piscivorous Chinese perch are rather monotypic compared to omnivorous fish (Chinese perch feed solely on live prey fish [49]). Although the genetic evidence was indirect, we assumed that the group ζ OR genes might be involve in recognizing compounds that come from non-fish. Our study awaits a function verification test, to confirm this group functional feature.

ORs are GPCRs that mostly contain seven transmembranes. Previous studies have revealed that some conserved amino acid motif features were found in the teleost ORs [34,40,48]. We generally obtained the same results. It was revealed that the components and positions of the ligand-binding residues of fish ORs remain highly conserved, despite the complicated aquatic environments. The motif V, located at EC1, is a potential position for binding multifarious compounds, and it was highly varied, to better accommodate thousands of odor molecules.

The ratio of dN/dS, also called Ka/Ks, is usually considered for the evaluation of the selection pressure experienced on genes during evolution. These values for Chinese perch OR groups were obviously above the average of 0.11, as calculated for 1880 human–rodent orthologous gene pairs [50], and similar to the values obtained from catfish and cichlid ORs [34,48]. We found that none of the OR groups exhibited positive selection. Rather, with average dN/dS ratios <1, these OR groups all appeared to be under negative or purifying selection. Interestingly, group η displayed the highest average dN/dS ratios compared to the others. The observation that group η was, in general, under less purifying selection than the other groups of OR genes was consistent with the possibility that they may have adapted to become more divergent, which has been investigated in the orthologs of group η, family H, by Alioto and Ngai [40]. 

### 4.3. Expression Levels of OR Genes in Chinese Perch OE

OR gene expression patterns in the OE have been reported for many animals. In the mouse, only a small part of the OR genes are expressed in the OE, and the remainder are transcriptionally inactive [51]. In addition, group ε and ζ are not detected in gene expression in the OE of large yellow croaker [21]. This is the reason for why only 64 OR genes were found to be expressed at 30 dph and adult OE, and why almost 50% of OR genes were not detectable in Chinese perch. Compared to zebrafish, the widespread lower expressions of OR genes were found at both 30 dph and at adulthood, suggesting that the functions of OR genes become less crucial in the Chinese perch, which has likely results from the adaption of relying less on olfaction in foraging. We found that several pseudogenes were still transcribed in the Chinese perch OE. Other researchers also found a similar result in the mouse olfactory epithelium [51].

### 4.4. Adaptive Evolution of OR Genes in Chinese Perch

The existence of unique or common OR genes across different species reflects the diversification or maintenance of orthologous genes from common ancestors during the evolution of the species [39]. Compared with four other Percomorpha fish (*n* = 27 to 45), Chinese perch had the least number of subfamilies (*n* = 22), but it had the largest number of OR genes in one subfamily (*n* = 23). These results suggested that the specific subfamily expanded, while some others degenerated over the evolution of Chinese perch. The most exciting thing was that Chinese perch specifically lost subfamilies 56 and 66. The OR genes of subfamily 66 were orthologous to human OR51E2, according to the HORDE database. OR51E2 could be activated by the odorant β-ionone, which is an isoprenoid that is widely found in plants and plant products as a degradation product of carotenoids [52,53]. Orthologous OR genes among different species in the same subfamily might recognize similar odorant substances, since it had been reported that OR genes sharing more than 60% in their sequence homology bind to odorants with similar chemical structures [39,54,55]. As such, Chinese perch, which obligately forages for live prey, specifically lost those receptors that recognized plant odors, perhaps due to the absence of plants throughout its entire life. From early on, the evolution of the OR repertoire was adapted to olfaction-independent feeding behavior, and a monotonous diet of live prey in Chinese perch. Although we found possible genetic evidence for a reduction in the reliance on olfaction in Chinese perch while feeding, future studies on olfactory function in different life activities in Chinese perch and other vision-dependent fish would provide a better understanding of the roles of smelling in Chinese perch. 

## 5. Conclusions

This study provides the first comprehensive and systematic analysis of the OR repertoire in Chinese perch. In total, 152 Chinese perch OR genes were identified and distributed on chromosomes in clusters. The gene features, conserved motifs, and phylogenetic relationship analyses further supported the evolutionary dynamics of Chinese perch. Finally, the expression profiles of these OR genes in different developmental stages were identified by RNA-seq analysis, and they showed the decrease expression level during the development. The results indicated that Chinese perch OR genes reduced in diversity, and specifically lost two families, in order to adapt to the reduction of olfaction in foraging. These results provide an essential foundation for further functional studies on the characteristics of chemosensory responses in Chinese perch.

## Figures and Tables

**Table 1:** A summary description of the olfactory receptor (OR) genes in Chinese perch.

Chromosome Number	No. of Functional Genes	No. of Pseudogenes (%)		Total	No. of Clusters
LG4	8	2	(20)	10	1
LG6	1	0	(0)	1	1
LG9	37	12	(24)	49	2
LG19	40	4	(9)	44	2
LG22	33	2	(6)	35	2
LGUN	4	9	-	13	-
Total	123	29	(19)	152	8

**Table 2:** Number of functional OR genes and pseudogenes (in parentheses) for each group among different fishes.

Species	α	β	γ	δ	ε	ζ	η	Non-OR	Total	Reference
Spotted gar	3	18	34(10)	55(2)	3	34(2)	8	1	156(14)	This study
Zebrafish	0	4(2)	1	62(7)	12(1)	37(4)	38(7)	0	154(21)	[11]
**Chinese perch**	**0**	**6(1)**	**1**	**74(13)**	**8**	**13(13)**	**22(2)**	**1**	**123(29)**	This study
Seabass	0	5(1)	1	79(2)	6(1)	9	31	0	131(4)	This study
Tongue sole	0	1	0	62(4)	6	10	16	1	96(4)	This study
Stickleback	0	1	0(3)	71(41)	4	18(4)	8(4)	0	102(52)	[11]
Fugu	0	1	0	30(25)	2(1)	4(2)	10(11)	0	47(39)	[11]

**Table 3:** Number of species-common or special OR genes among five Percomorpha fish OR repertoires.

	Number of OR Genes Belonging to Common Subfamilies in the Species				
	Sich	Dila	Cyse	Taru	Gaac
Sich, Dila, Cyse, Taru, Gaac	15	11	11	5	16
Sich, Dila, Cyse, Taru	3	3	1	1	-
Sich, Dila, Cyse, Gaac	16	21	8	-	10
Sich, Dila, Taru, Gaac	35	22	-	4	5
Sich, Cyse, Taru, Gaac	3	-	4	2	2
Dila, Cyse, Taru, Gaac	-	5	6	2	14
Sich, Dila,	28	23	-	-	-
Sich	7	-	-	-	-
Dila	-	13	-	-	-
Cyse	-	-	50	-	-
Taru	-	-	-	9	-
Gaac	-	-	-	-	22

“Sich”, “Dila”, “Cyse”, “Taru”, and “Gaac” represent Chinese perch, European seabass, Tongue sole, fugu, and stickleback, respectively.

## Supplementary Materials

The following are available online at https://www.mdpi.com/2073-4425/10/2/178/s1, Supplementary data 1: Chinese perch genomic contigs containing OR gene clusters and the annotation file. Supplementary data 2: The Chinese perch OR repertoire in the genome, Supplementary data 3: The OR amino acid sequences of six other fish, used for phylogenetic analysis, Supplementary data 4: Comparison of OR-functional gene identities among five Percomorpha fishes, Supplementary data 5: The numbers of OR genes distributed in each family and subfamily, Supplementary data 6: Comparison of the structures of functional OR genes among five Percomorpha fishes, Supplementary data 7: *N*-glycosylation sites as predicted by the NetNGly Server for Chinese perch OR, Supplementary data 8: The FPKM values of OR genes at different developmental stages in Chinese perch olfactory epithelium (OE).
